# Early IL-6 response in sepsis is correlated with mortality and severity score

**DOI:** 10.1186/cc11972

**Published:** 2013-03-19

**Authors:** P Srisangthong, A Wongsa, P Kittiworawitkul, A Wattanathum

**Affiliations:** 1 Phramongkutklao Hospital, Bangkok, Thailand

## Introduction

IL-6, a proinflammatory cytokine, is synthesized from fibro-blasts, T lymphocytes, endothelial cells and monocytes. It serves as an important mediator during the acute phase response to inflammation in sepsis. We hypothesized that the plasma IL-6 is correlated with mortality and severity scores in critically ill patients with sepsis.

## Methods

We conducted a prospective study of plasma IL-6 level at the initial phase of sepsis and the risk of mortality. A total of 203 patients with sepsis, who were admitted to the medical ICU at Phramongkutklao Hospital, Bangkok during January to December 2011, were analyzed. Serum IL-6, C-reactive protein (CRP), and lactate were measured within the first 24 hours of ICU admission. Severity scores (APACHE II, SAP II, and SOFA scores) were measured. The primary outcome variable was 28-day all-cause mortality.

## Results

We found that the overall 28-day mortality was 46% (93 out of 203 patients). There was a significantly positive correlation between mortality rate and plasma IL-6 (survivors vs. nonsurvivors; 74 (4.4 to 1,718) vs. 206 (19 to 5,000) pg/ml, *P <*0.05), lactate (survivors vs. nonsurvivors; 1.65 (0.7 to 11.61) vs. 2.47 (0.94 to 19.13) mmol/l, *P <*0.05), but not CRP levels (*P *= 0.24). Compared with the patients with plasma IL-6 <100 pg/ml, septic patients with IL-6 levels ≥100 were associated with an increased 28-day mortality with the odd ratio of 2.99 (95% CI 1.42 to 6.29, *P <*0.05). We also found that plasma IL-6 levels were well correlated with APACHE II (*P <*0.05), SAPS II (*P <*0.05), and SOFA (*P <*0.05) scores.

## Conclusion

The initial phase plasma IL-6 levels were correlated with severity and mortality in critically ill patients with sepsis.
